# Electroacupuncture ameliorates incisional pain via suppressing IL-33 signaling-related macrophage infiltration and ROS overproduction in incised skin

**DOI:** 10.1186/s13020-025-01273-0

**Published:** 2026-01-09

**Authors:** Ruoyao Xu, Kaige Zheng, Yushuang Pan, Peiyi Li, Muyan Chen, Huimin Nie, Boyu Liu, Yan Tai, Xiaofen He, Junying Du, Jianqiao Fang, Guihua Tian, Boyi Liu

**Affiliations:** 1 Department of Neurobiology and Acupuncture Research, The Third Clinical Medical College, Key Laboratory of Acupuncture and Neurology of Zhejiang Province, Zhejiang Chinese Medical University, Hangzhou, China; 2 Academy of Chinese Medical Sciences, Zhejiang Chinese Medical University, Hangzhou, China; 3 Department of Chinese Medicine, Beijing Friendship Hospital, Capital Medical University, Beijing, China

**Keywords:** Acupuncture, IL-33, Macrophage, ROS, Incisional pain

## Abstract

**Background:**

Postoperative pain develops after surgical operation. Inadequately managed postoperative pain is oftentimes associated with deterioration in life quality and delayed rehabilitation. Clinical studies confirmed analgesic effects of acupuncture on patients with postoperative pain. We hereby explored mechanisms underlying how acupuncture alleviates postoperative pain.

**Methods:**

A mouse model of skin incision-induced pain was established to mimic postoperative pain condition. Electroacupuncture (EA) or sham EA was administered at ST36 and BL60 acupoints in model mice. A combination of RNA-Sequencing (RNA-Seq), immunostaining, biochemical assay, in vivo imaging and behavioral test were applied for mechanism investigations.

**Results:**

2/100 Hz EA intervention ameliorated mechanical allodynia and improved cumulative pain of incisional pain model mice. Sham EA exerted no obvious analgesic effect. Incisional pain model mice develop gait impairment, which was improved by EA intervention. RNA-Seq identified EA significantly reduced *Il33* gene overexpression in the incised skin tissues of model mice. IL-33 was produced from keratinocytes upon skin incision. EA reduced IL-33 overproduction from keratinocytes, resulting in less macrophage infiltration and less ROS accumulation in the incised skin. Adoptive transfer of macrophages into the incised tissue abrogated the ameliorative effects of EA on macrophage infiltration as well as ROS accumulation in incised skin and further reversed EA-induced analgesia on incisional pain model mice. Additionally, EA intervention did not affect skin wound healing process.

**Conclusion:**

These results demonstrate that EA ameliorates incisional pain via suppressing IL-33 overproduction in incised skin tissue. EA-induced suppression of IL-33 overproduction results in less macrophage infiltration and ROS accumulation that contribute to analgesia. Our work helps to support EA as an alternative and green therapy for postoperative pain management.

**Supplementary Information:**

The online version contains supplementary material available at 10.1186/s13020-025-01273-0.

## Introduction

Postoperative pain develops after a surgical operation and is a critical determinant of the patient’s outcome [1, 2]. It is reported that over 40% of patients report moderate or severe pain at the surgical site within 24 h of surgery [2, 3]. Inadequate analgesia for postoperative pain has been consistently related with adverse clinical trajectories, including prolonged hospitalization, elevated complication risks, and suboptimal recovery profile [1]. This condition remains a significant challenge for perioperative care providers, especially pain specialists [2]. Current analgesia predominantly rely on opioid-based therapies and nonsteroidal anti-inflammatory drugs (NSAIDs) as cornerstone interventions [4–6]. While these pharmacological approaches can provide essential analgesic efficacy, their application is increasingly tempered by emerging concerns over opioid-related adverse effects and NSAIDs-induced organ toxicities [4, 5]. Therefore, identifying alternative analgesic approaches with less adverse effects for postoperative pain has important clinical significance.

Tissue inflammation represents a major contributing factor to the pathogenesis of postoperative pain [7, 8]. Following surgery, a diverse array of immune cells, such as neutrophils, monocytes, and macrophages, migrate to the injured tissues [9]. Upon arrival, these cells secrete a broad spectrum of pro-inflammatory cytokines and chemokines, which subsequently mediate neuro-immune interactions through bidirectional signaling. On one hand, these molecules play important roles in helping to constrain tissue injuries or infections [10]. On the other hand, some of these molecules can either activate or sensitize nociceptors, thus triggering pain and peripheral sensitization [7]. Additionally, upon skin incision, keratinocytes also secrete a panel of inflammatory mediators such as ATP, NGF and IL-33, etc. [11–13]. These substances may directly target receptors expressed by sensory neurons or attract immune cells to participate in postoperative pain. Our recent work identified IL-33 is produced from keratinocytes and released into extracellular space to attract macrophage via receptor ST2 upon skin incision [12]. The infiltrated macrophages then release large amounts of ROS, which subsequently activate TRPA1 in sensory neurons innervating incisional skin to produce pain [12]. Therefore, the dynamic crosstalk between immune responses and sensory neuron activation plays a critical role in the generation and persistence of postoperative pain condition.

Acupuncture has been widely used for pain management in the worldwide [14]. Electroacupuncture (EA), which integrates modern electrical stimulation technology with traditional acupuncture technique, has been extensively adopted in clinical practice for managing diverse pain conditions [15]. Clinical trials and meta-analyses conclude that acupuncture or EA is an effective method for alleviating postoperative pain and reducing the dosage of analgesics [16, 17]. These work have indicated the effectiveness of acupuncture for managing postoperative pain. When using incisional pain model to emulate postoperative pain states, EA has been mechanistically validated to attenuate incisional pain via multi-target modulation in spinal and brain levels. These mechanism include upregulating GABA, cannabinoid receptor type 1 (CB1R) and glutamine transporter GLT-1 expression, while downregulating TNF-α signaling in spinal dorsal horn, etc., thereby suppressing nociceptive pathway at spinal level [18–21]. More recently, it is found that the locus coeruleus noradrenergic-spinal projection contributes to EA-induced analgesia in postoperative pain via triggering spinal noradrenaline release [22]. Prior studies from us and others have demonstrated that EA can mitigate local inflammation to exert analgesic effects on inflammatory pain conditions [23–26]. However, it still remains largely unknown whether EA may exert analgesia through modulating local inflammation in the incised tissues under postoperative pain condition.

Hereby, we established a mouse model of skin incision-induced pain model and explored mechanisms underlying analgesic effects of EA with the focus on whether EA might affect IL-33-mediated nociceptive signaling in local incised skin tissues. Our results demonstrate that EA intervention can reduce IL-33 overproduction from keratinocytes upon skin tissue incision, resulting in less macrophage infiltration and less ROS accumulation in local incised skin tissues. These effects may contribute to EA-induced analgesia on incisional pain model mice.

## Methods and materials

### Animals

Male BALB/c mice (around 6 weeks; 18–20 g) were bought from Shanghai Laboratory Animal Center. All animals were house in Laboratory Animal Center of Zhejiang Chinese Medical University with standard environmental condition (12 h light–dark cycle and 24 °C). Food and water are provided ad libitum. The mice were randomly allocated and 5 mice were housed per cage.

### Skin incisional pain model establishment

Mice were anesthetized with 3.5% isoflurane in an induction chamber and maintained with 1.5% isoflurane via a nose cone. The plantar area of hind paw was disinfected using 70% alcohol before operation. A 5 mm longitudinal incision with made with a No.11 blade through the skin and fascia of the plantar foot (2 mm away from the edge of the heel). To further study deep tissue incision-induced pain, the underlying muscle was elevated with a curved sterile forceps, leaving muscle origin and insertion intact, as previously described [27]. After the operation, the skin was sealed with a single mattress suture of 6–0 nylon suture. The mice then underwent recover from anesthesia on a heating pad. Sutures were removed 3 day after the incision. The control mice (sham operation) receive the same anesthesia and disinfection as above but without incision.

### Mechanical allodynia

Individually, mice were put into a transparent Plexiglas chamber set on an elevated mesh floor. They underwent a 30-min habituation period prior to formal testing. To measure mechanical allodynia, a set of von Frey filaments ranging from 0.07 to 2 g were employed. The filaments were applied vertically to the plantar surface of the mouse's hind paw, starting with the thinnest fiber and increasing in thickness.

The minimum force of each monofilament that led to the mouse retracting its hind paw was regarded as the withdrawal threshold. For each mouse, a von Frey hair was used to stimulate the plantar surface of the right hind paw near the incision. The stimulation was performed 5 times at 10-s intervals and there was a 10-s break between successive stimuli. The stimulation began with a force of 0.07 g and the force was gradually increased. As previously reported [28, 29], the threshold was identified when the paw withdrawal response occurred in more than 3 out of the 5 applications.

### Cumulative pain score

A cumulative pain score was employed to evaluate non-evoked pain behaviors as reported [30]. Prior to the test, the unrestrained animals were put on an elevated stainless steel mesh floor (with a 4 × 4 mm grid) inside a clear plastic cage. They were then allowed 30 min to adapt to the experimental room environment. An angled magnifying mirror was used to observe the incised foot. Each animal was carefully watched for 1 min, and this observation was repeated every 5 min over a 1-h period. Based on the position of the foot during most of the 1-min scoring interval, a score of 0, 1, or 2 was assigned. A score of 0 was given when the foot was fully bearing weight, which was indicated by the wound being blanched or distorted by the mesh. A score of 2 was recorded if the foot was completely off the mesh. If the wound area touched the mesh without blanching or distortion, a score of 1 was assigned. For each foot, the total of the 12 scores (ranging from 0 to 24) during the 1-h session was calculated.

### Electroacupuncture (EA) intervention

The mice were immobilized with a restrainer. Acupuncture needles (0.16 × 7 mm diameter) were inserted at a depth of 4 mm into bilateral Zusanli (ST36, 5 mm lateral to the anterior tubercula of the tibia) and Kunlun (BL60, at the ankle joint level and between the tip of the external malleolus and calcaneus) acupoints. Bilateral EA stimulation protocol was selected based upon our previous studies [24, 31, 32]. In these studies, we achieved satisfied analgesic effect and anti-inflammatory effect on inflammatory pain model animals. Animals in sham EA groups were treated with needle insertion subcutaneously but without electric stimulation. All other groups of mice received the same immobilizing treatment as EA or sham EA group. The needles were connected with HANS-200A acupuncture point nerve stimulator (HANS.200A, Ji sheng Co., Ltd., Nanjing, China) using alligator clips with electrical wires. Interventional parameters: 2, 100 or 2/100 Hz frequency, 1.0 mA stimulating intensity, 30 min intervention/session. The EA/sham EA interventions were carried out at −1, 0.25, 1, 2, 3, 4, 5, 6, and 7 day time points, respectively.

### Gait recording and analysis

This method was used to measure gait behavior affected by incisional pain in mice. The DigiGait imaging system (MouseSpecifics, Inc., USA) was employed to assess locomotor adaptations induced by incisional pain in rodents, following established protocols [33]. The mouse was put on a flat and transparent treadmill, which was operated on a constant speed (18 cm/s). A video camera was located underneath the apparatus to record gait of mice while it was running, and captured images of the illuminated area of each paw. The animals were allowed to run on the treadmill for a period of 20 s and a consecutive 5 strides were averaged per animal and used for analysis. Parameters including paw area, stance, stride and swing were calculated via the software.

### Western blot

To measure the protein expression of IL-33, skin samples were harvested at 1 day after the incision. Samples were homogenized in RIPA buffer [50 mM Tris (pH7.4), 150 mM NaCl, 1% Triton X-100, 1% sodium deoxycholate, sodium orthovanadate, 0.1% SDS, EDTA, sodium fluoride, leupeptin, and 1 nM PMSF], then centrifuged at 12,000 rpm for 10 min at 4 °C and the supernatant was then collected. The protein concentration was determined using BCA method according to the kit’s instruction (Thermo Fisher, USA) and 15 μg protein was loaded in each lane. Protein was loaded and separated by SDS‐PAGE and electrophoretically transferred onto PVDF membranes. The membranes were blocked with 5% non-fat milk in TBST solution for 1 h at room temperature, and then the membranes were incubated with primary antibodies: IL-33 (1:500, #AF3626, R&D Systems, USA) overnight at 4 °C. Subsequently, the immunoblots were incubated with the secondary antibody anti-gout IgG (1:2000, #31402, Thermo Fisher, USA) for 1.5 h at room temperature. β-actin (1:5000, #ab20272, Abcam, UK) was used as reference control. The gel images were captured by FluoChem R (Biotechne, USA). Quantitative analysis was performed with ImageJ (NIH, USA).

### Immunostaining

The mice were deeply anesthetized with isoflurane, and were perfused through the ascending aorta with 0.9% saline followed by 4% fresh paraformaldehyde in 0.01 M PBS. After perfusion, the ipsilateral skin were removed and post‐fixed in 4% paraformaldehyde for 4–6 h (4 °C) before transferring to 15% and 30% sucrose for dehydration. Samples embedded in a frozen microtome (Thermo NX50, Thermo Fisher, USA) were cut into frozen sections with the thickness of 12 μm. Then samples were mounted onto gelatin-coated glass slides for immunofluorescence. The tissues were first blocked with 5% donkey serum in TBST for 1 h at 37 °C, then incubated with the following primary antibodies at 4 °C overnight: anti-IL33 (1:200, #AF3626, R&D Systems, USA), anti-keratin14 (1:200, #ab119695, Abcam, UK), anti-Iba1 (1:500, #ab178846, Abcam, UK), anti-8-OHdG (1:500, #ab62623, Abcam, UK). After washing, the tissues were incubated with corresponding secondary antibodies (Cy3-, Cy5-, or FITC-conjugated) for 1 h at 37 °C after washing in the dark. Images were capture by Axio Imager M2 microscope (Zeiss, Germany). Fluorescence images were taken via the ZEN system (Zeiss, Germany). For quantification, a uniformed microscope setting was maintained throughout image capture session as previously described [34]. Fluorescence intensity was obtained from ImageJ (NIH, USA). When calculating the normalized fluorescence intensity, the fluorescence intensity values from each group were normalized with the value from the control group or the designated group as indicated in the figures and corresponding figure legends.

### RNA-Seq and data processing

The skin tissues were collected 1 day after the incision, diced and stored in RNAlater (Thermal Fisher Scientific, USA). Total RNA from Sham, INC and INC + EA group was extracted using Trizol reagent (Thermal Fisher Scientific, USA). RNA quality and purity were validated by TapeStation (Agilent, USA) and NanoDrop (Thermo Fisher Scientific, USA). Only RNA samples showing RNA Integrity Number ≥ 8.0 and A260/230 ≥ 1.5 were used for RNA-Seq. The samples were sequenced by BGISEQ-500 from BGI Group (Shenzhen, China). Raw sequencing reads were aligned to mouse genome (mm10). Differential expression analyses were performed with R and Bioconductor packages of edgeR and limma voom as reported in our previous studies [35, 36]. The threshold required for the genes to be considered significantly changed was as follows: q ≤ 0.05 and absolute value of |log_2_ (Fold Change)|≥ 0.58.

### qPCR

This method was used to examine gene expression changes. Total RNA from ipsilateral hind paw skin tissue was extracted by TRIzol reagent (Thermo Fisher, USA). 1,000 ng of total RNA was reversely transcribed with PrimeScript RT Reagent Kit (Takara Bio Inc., Japan) into cDNAs. qPCR was performed using TB Green Premix Ex Taq II (Takara Bio Inc, Japan) as the master kit with CFX96 Real-Time System (Bio-Rad Laboratories Inc., USA). β-actin gene (*Actb*) was used as an internal reference gene. Each reaction was performed in triplicates and normalized to *Actb* gene expression. The cycle threshold (CT) value of each well was deduced using CFX96 Real-Time System software and the average of the triplicates was calculated. The ΔΔCT method was utilized to determined relative quantification [37, 38]. The detailed information regarding the sequences for the primer sequence (from 5ʹ to 3ʹ) was as follows:

*Actb* forward: 5'-GTGCTATGTTGCTCTAGACTT CG-3',

*Actb* reverse: 5'-ATGCCACAGGATTCCATA CC-3';

*Il33* forward: 5’-CAGAAGACCAAAGAATT CTGCC-3’,

*Il33* reverse: 5’-CATGCTTGGTACCCGA TTTTAG-3’;

### In vitro ROS assay

This method was used to evaluate ROS level in an in vitro setting. The detailed protocols of oxidative stress biomarkers examination have been documented in our previous publication [39]. Ipsilateral hind paw skin tissues from each group were collected 3 d after incision. All samples were chopped and centrifuged and then the supernatant was collected for corresponding biochemical assays. The collected supernatants were then analyzed by means of commercially available kits for superoxide dismutase (SOD), reduced glutathione (GSH) (Nanjing Jiancheng Bioengineering Institute, China), malondialdehyde (MDA) and hydrogen peroxide (H_2_O_2_) (Beyotime Biotechnology, China) according to the instructions. The absorbance was determined with SpectraMax M4 (Molecular Devices, USA) microplate reader at specified wavelength and the data was derived and analyzed using SoftMax Pro software (Molecular Devices, USA).

### In vivo ROS imaging

This method was used to monitor ROS changes in the incised skin tissue in an in vivo setting. 3 d after incision, the mice were injected (intravenously, i.v.) with chemiluminescence ROS probe L-012 (25 mg/kg, Tocris, USA), as described previously [40]. Mice were anesthetized with isoflurane, then placed on the stage of an IVIS Lumina LT in vivo imaging system (PerkinElmer, USA) and the fluorescence imaging was performed 5 min after the injection. The luminescence signal intensities were quantified with Living Image software (PerkinElmer, USA).

### Macrophage adoptive transfer

This method was used to study the contribution of macrophages to the nocifensive behavior and oxidative stress in INC model mice. Thioglycollate-elicited macrophages (TPMs) were generated by injecting the mice (i.p.) with 1 ml 4% Brewer’s thioglycollate medium (Sigma, USA) for 3 consecutive days, adding up to 3 ml in total as previously reported [40]. Mice were euthanized 1 d after the last injection. Peritoneal cavity cells were harvested by lavage, and cultured in DMEM plus 10% FBS, 100 U/mL penicillin and 100 μg/mL streptomycin. The TPMs of the donor mice were injected (i.pl.) into hind paws of receptive mice at a dose of 5 × 10^4^ cells per mouse 2 h before the EA intervention at the 0.25 d time point.

### Wound healing assessment

For assessing wound closure following the plantar incision, the width of each incision site was measured every day and the photo of the incision site was taken on 1, 3, 5, and 7 days after the surgery. Changes in the wound areas were expressed as the width of the incised site as before [12].

### Data processing and statistical analysis

The raw data were processed into respective presentation forms as in the figures of this study via GraphPad Prism 8 (GraphPad Software Inc., USA) and Adobe Illustrator CC 2017 (Adobe, USA). The GraphPad Prism 8 was conducted for statistical analysis in this study. Data in bar graphs were expressed as mean ± SEM. Student’s *t*-test was used for comparisons between two groups. One-way or two-way ANOVA followed by Tukey’s post hoc test was used for comparison among groups ≥ 3. ANOVA with repeated measures was taken if necessary. In cases when the data was non-normally distributed (tested by Kolmogorov–Smirnov test), a nonparametric test (e.g. Mann–Whitney test) was used for analysis. Comparison is considered significantly different if p < 0.05.

## Results

### Electroacupuncture intervention relieves pain and improves gait impairments in a mouse model of incisional pain

**Fig. 1:**
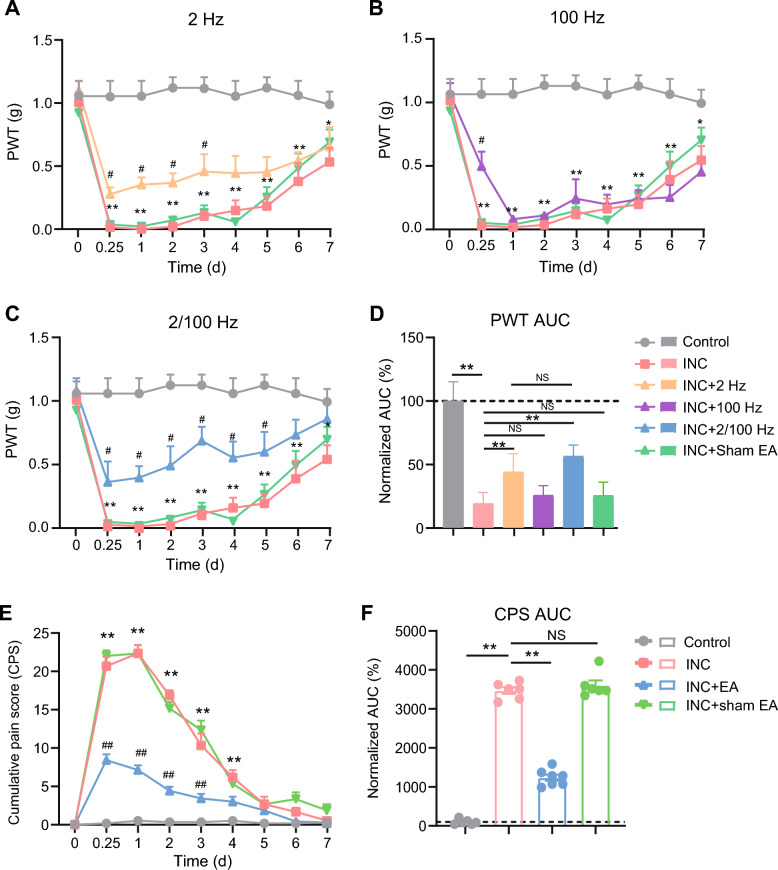
The screening of optimal EA frequency for controlling incisional pain. **A**–**C** Overlaid time courses showing the paw withdraw threshold (PWT) changes of control, incisional pain model (INC), incisional model with EA or sham intervention (INC + EA or INC + sham EA) groups of mice. 2, 100 or 2/100 Hz EA was applied through panel A-C as indicated, respectively. ^**^p < 0.01, ^*^p < 0.05 vs. control group. ^#^p < 0.05 vs. INC group. Curves showing control, INC and INC + shamEA groups were shared from panel A-C. **D** Summary of normalized area under the curve (AUC) analyses of the curves as shown in panel A-C. The values from control group were taken as 100% and all other groups were normalized afterwards. n = 6–7 mice/group. ^**^p < 0.01. *NS* no significance. **E** Time courses showing the changes in cumulative pain scores (CPS) of 4 groups of mice as indicated. ^**^p < 0.01 vs. control group. ^##^p < 0.01 vs. INC group. **F** Summary of normalized AUC analyses of the curves shown in panel **E**. The value from control group was normalized as 100%. n = 6–7 mice/group

A mouse model of skin incision-induced pain was established to evaluate the analgesic effect of electroacupuncture (EA). After the incision was made, the mice developed obvious signs of mechanical allodynia, as demonstrated by the significantly decreased paw withdraw threshold (PWT) (Fig. 1A). The mechanical allodynia lasted for over 7 days (Fig. 1A). It is known that distinct EA stimulation frequency could result in different analgesic effect. We first explored the optimal frequency of EA for alleviating mechanical allodynia of incisional pain model mice. We started by examining 2, 100, or 2/100 Hz EA on bilateral ST36 and BL60 acupoints of mice, respectively. Sham EA, with shallow insertion of needles into ST36 and BL60 but with no current stimulation, was adopted as the negative control as before [41]. EA or sham EA was applied daily after the incision was made. We found that 2 Hz EA moderately improve the mechanical allodynia when compared with incisional (INC) pain group mice (Fig. 1A). 100 Hz only produced analgesia during the very beginning of the treatment session (Fig. 1B). In contrast, 2/100 Hz EA could produce a persistent alleviation of the mechanical allodynia of the incisional pain model group mice, which lasted over 5 days (Fig. 1C, D). In contrast, sham EA had no obvious analgesic effect compared with incisional pain model group (Fig. 1C, D). Therefore, in our following studies, we adopted 2/100 Hz as the optimal parameter of EA for incisional pain intervention in animal model.

In addition to evoked pain behaviors (e.g. mechanical allodynia we here tested), incisional pain model mice also displayed non-evoked pain behaviors [30], a phenomenon similar with humans. We then studied the effect of EA on non-evoked pain of incisional pain model mice that was evaluated by the cumulative pain score (CPS) calculation. Mice with incisional pain developed a robust increase in CPS compared with the control group (Fig. 1E, F). We found that 2/100 Hz EA could significantly reduce CPS in incisional pain model mice, whereas sham EA had no obvious effect on CPS (Fig. 1E, F).

**Fig. 2:**
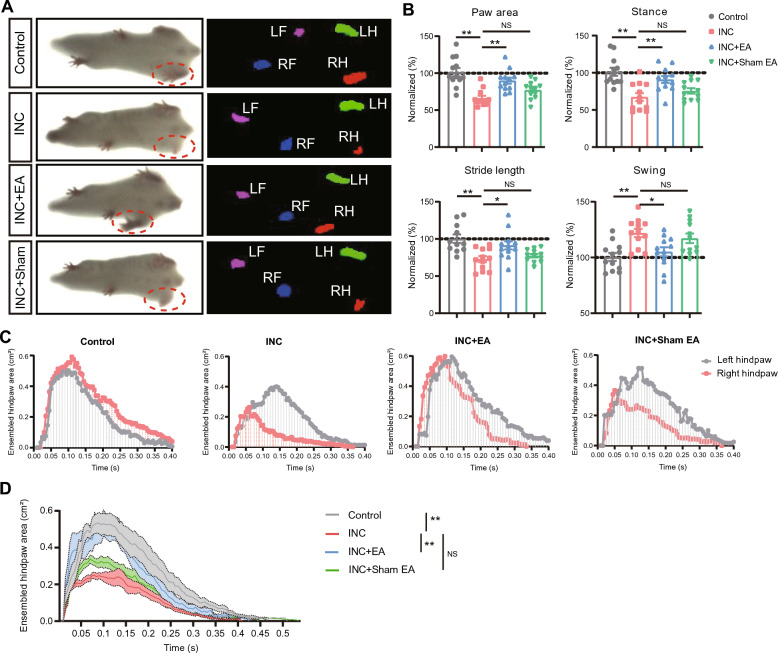
EA intervention improves gait impairment of incisional pain model mice. **A** Representative photos showing the mice monitored and analyzed through the gait analyzing system. Four groups (control, INC, INC + EA and INC + sham EA) were included for gait analysis. Left picture: instant recordings of mice. Right picture: gait analysis. LH: left hind paw; RH: right hind paw; LF: left forepaw; RF: right forepaw. **B** Summarized gait parameters including paw area, stance, stride length and swing of 4 groups of mice one day after incision. n = 11–12 mice/group. Data from the control group was normalized as 100%. **C** Typical traces illustrating the dynamic change of ensemble hind paw area of the right compared with left hind paw one day after incision. **D** Summary of the dynamic changes in ensemble hind paw area (right) obtained from 4 groups as in panel C. n = 11–12 mice/group. ^*^p < 0.05, ^**^p < 0.01. *NS* no significance

Movements can exacerbate incisional pain, which can restrict patients’ mobility, daily activities, and even postsurgical rehabilitation participation [42]. Our recent work revealed incisional pain model mice developed significant gait impairments [12]. We next evaluated whether EA may improve the gait impairments of incisional pain model mice. One day after the incision, we found that the incisional pain model mice began to develop obvious signs of impaired gait in the affected hind paw compared with control mice in terms of paw area, stance, stride length and swing (Fig. 2A, B). After 2/100 Hz EA intervention, the impaired gait of incisional pain model mice were significantly improved. In contrast, sham EA showed no obvious effect (Fig. 2A, B). We then took a closer look at the hind paw print dynamics. A more refined spatiotemporal analysis of paw print dynamics revealed that incisional pain mode mice exhibited abnormal dynamic changes in ensemble area compared with control mice (Fig. 2C, D). In contrast, EA-treated mice demonstrated significant improvement in the ensemble area compared with control group (Fig. 2C, D). However, sham EA-treated mice showed no obvious change when comparing with the incisional pain model mice (Fig. 2C, D). These data thus demonstrates that EA intervention can produce persistent analgesia and successfully improve gait impairment in incisional pain model mice.

### EA intervention reduces IL-33 overproduction in skin tissues of incisional pain model mice

**Fig. 3:**
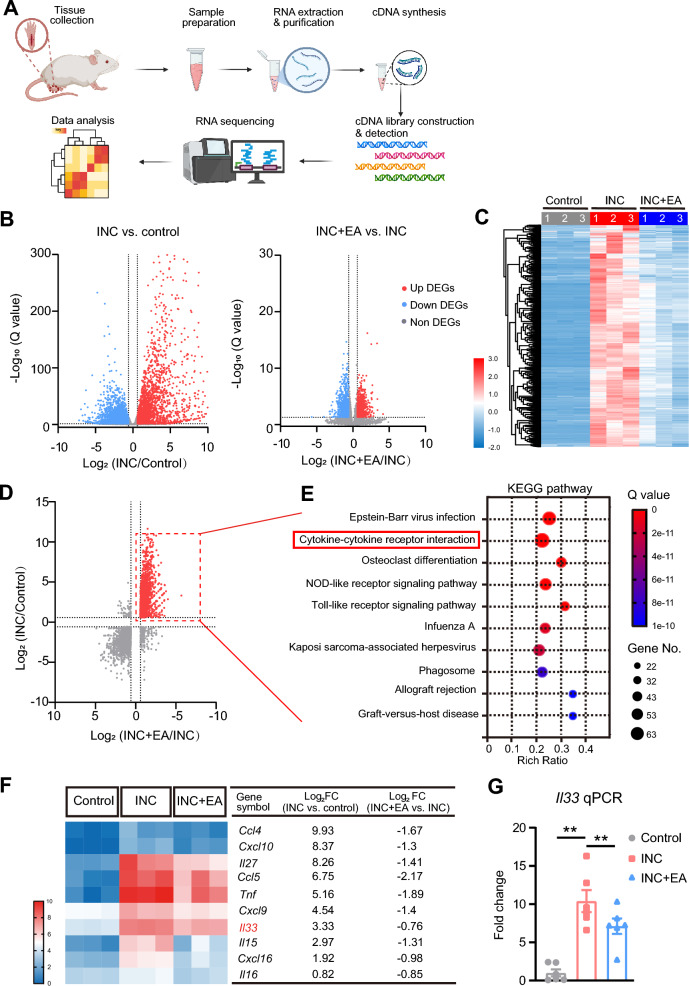
RNA-Seq and bioinformatics analysis of DEGs in the incised skin of incisional pain model mice after EA intervention. **A** Cartoon showing the workflow of RNA-Seq and bioinformatics analysis included in the present study. Picture was created with BioRender.com. **B** Volcano plot showing DEGs identified from incisional pain (INC) group vs. control group. n = 3 mice/group. **C** Volcano plot showing DEGs identified from INC + EA vs. INC group. n = 3 mice/group. **D** Scattered plot showing DEGs overlapped between INC vs. Control group and INC + EA vs. INC group. **E** KEGG analysis of the top 10 pathways enriched by the DEGs that showed upregulation in INC vs. Control group, whereas downregulated in INC + EA vs. INC group (as outlined by the dashed red box in panel **D**). **F** Heat map illustrating the top 10 DEGs identified from the “cytokine-cytokine receptor interaction” pathway via KEGG as shown by panel **E**. **G** qPCR validation of *Il33* gene expression in skin tissues of control, INC and INC + EA groups of mice. n = 5 mice/group

The incised skin produces a variety of inflammatory mediators that make important contributions to incisional pain mechanisms. To further explore how EA may alleviate pain in incisional pain model mice, we dissociated the skin tissues and performed bulk RNA-Sequencing (RNA-Seq) to explore gene expression changes in mice with or without EA treatment with special focus on inflammation-related pathway (Fig. 3A). Compared to the control group, our volcano plot analysis revealed that mice in incisional pain model group (INC group) exhibited 7,043 differentially expressed genes (DEGs), including 3,340 upregulated and 3,703 downregulated genes (Fig. 3B, left panel). Furthermore, when contrasted with incisional pain model mice (INC group), those INC model mice receiving EA treatment (INC + EA group) demonstrated 2,033 DEGs, characterized by 947 upregulated and 1,086 downregulated genes (Fig. 3B, right panel). The DEGs were further visualized using a heat map, as depicted in Fig. 3C. We then examined the overlapped DEGs between INC + EA/INC and INC/Control groups. Our analysis specifically focused on the DEGs exhibiting upregulation in INC/Control group but downregulation in INC + EA/INC group, as highlighted in the dashed red box (Fig. 3D). We performed KEGG analysis on this specific core set of DEGs that we identified. KEGG analysis indicated that EA intervention could significantly downregulate a series of signaling pathways involving in cytokine-cytokine receptor interaction, NOD-like receptor signaling pathway and toll-like receptor signaling pathway, etc. in the incised skin tissues from incisional pain model mice (Fig. 3E). Cytokine-cytokine receptor interaction is critically involved in mediating inflammation and pain mechanism. Within this core set of DEGs involved in this biological process, *Il33* gene appeared as one of the most significantly upregulated cytokine genes in INC group vs. control group, an elevation that was also suppressed following EA intervention (Fig. 3F). In our recent work, we unraveled a critical role of skin IL-33 in mediating incisional pain via recruiting pro-inflammatory macrophages that release ROS [12]. Thereafter, we assumed that EA might alleviate incisional pain via reducing IL-33 overproduction in the incised skin tissues. Then, we further validated this finding from RNA-Seq via performing qPCR. The qPCR results showed that *Il33* gene expression was significantly increased in the incised skin of incisional pain model mice (Fig. 3G), a result consistent with our recent finding [12]. Importantly, the increase in *Il33* gene expression was downregulated by EA intervention (Fig. 3G).

**Fig. 4:**
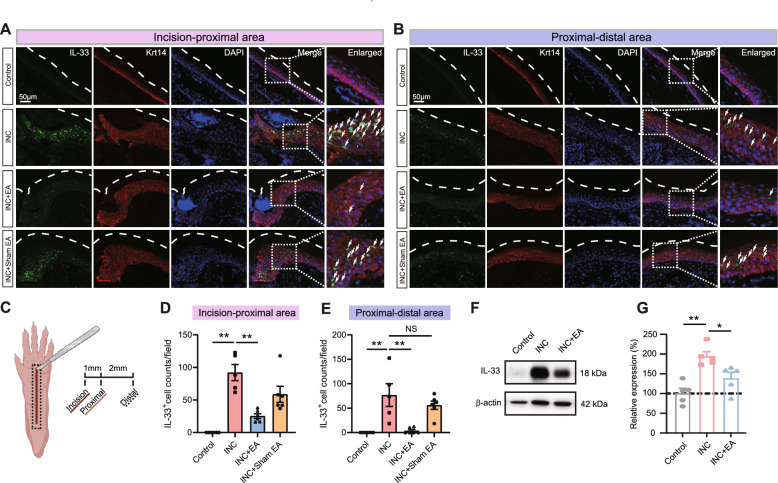
EA intervention reduces IL-33 overproduction in skin tissues of incisional pain model mice. **A**, **B** Immunostainings of IL-33 (green) and keratinocyte marker Krt14 (red) co-expression in the incised skin tissues of control, INC, INC + EA and INC + sham EA group of mice. The dashed white line outlines the stratum corneum of epidermis. DAPI was stained in purple color. Panel A was derived from skin tissues collected from “proximal-incision area” and panel B was derived from “proximal–distal area”, as shown by panel **C**. White arrows indicate IL-33^+^Krt14^+^ cells. Scale bar = 50 μm. **C** Schematic picture showing the skin incision and location of tissues being collected for analysis. **D**, **E** Summary results showing IL-33^+^ cell counts per observation field of skin tissue derived from proximal-incision area (panel **D**) and proximal–distal area (panel **E**). **F** Immunoblotting bands showing IL-33 expression in skin tissues of control, INC, INC + EA group. **G** Summarized results of immunoblotting as in panel F. n = 5 mice/group. ^*^p < 0.05 and ^**^p < 0.01

Our recent study revealed that IL-33 is overly produced by keratinocytes upon skin incision [12]. We next examined IL-33 protein expression in skin by immunostaining. We first examined skin tissues isolated from incision to the proximal area that includes the incised skin (Fig. 4A, C). Immunostaining showed that the number of IL-33 positively stained cells (IL-33^+^ cells) was significantly increased in incisional pain model mice compared with control mice (Fig. 4A). EA intervention significantly reduced the number of IL-33^+^ cells in incisional pain model mice, whereas sham EA had no such effect (Fig. 4A–D). Next, we focused on skin tissues isolated from proximal to distal area of the incisional site (Fig. 4B, C). Similarly, the number of IL-33^+^ cells was again significantly increased in incisional pain model mice vs. control mice (Fig. 4B, E). EA intervention significantly reduced the number of IL-33^+^ cells in incisional pain model mice, whereas sham EA didn’t show such effect (Fig. 4B, E). Furthermore, double immunostaining showed that IL-33 expression closely overlapped with cells co-stained with the keratinocyte specific marker keratin 14 (Krt14) in skin tissues isolated from both incision to proximal area and from proximal to distal area of the incisional site (Fig. 4A, B, as indicated by the white arrows). We further quantified IL-33 expression by immunoblotting. As shown in Fig. 4F, G, IL-33 expression was significantly upregulated in the incised tissues of incisional pain model mice compared with control group. EA intervention significantly reduced the IL-33 overexpression (Fig. 4F, G). Therefore, these results indicate that EA intervention could reduce IL-33 overproduction in skin tissues of incisional pain model mice.

### EA reduces macrophage infiltration and subsequent oxidative stress increase in incised skin of incisional pain model mice

**Fig. 5:**
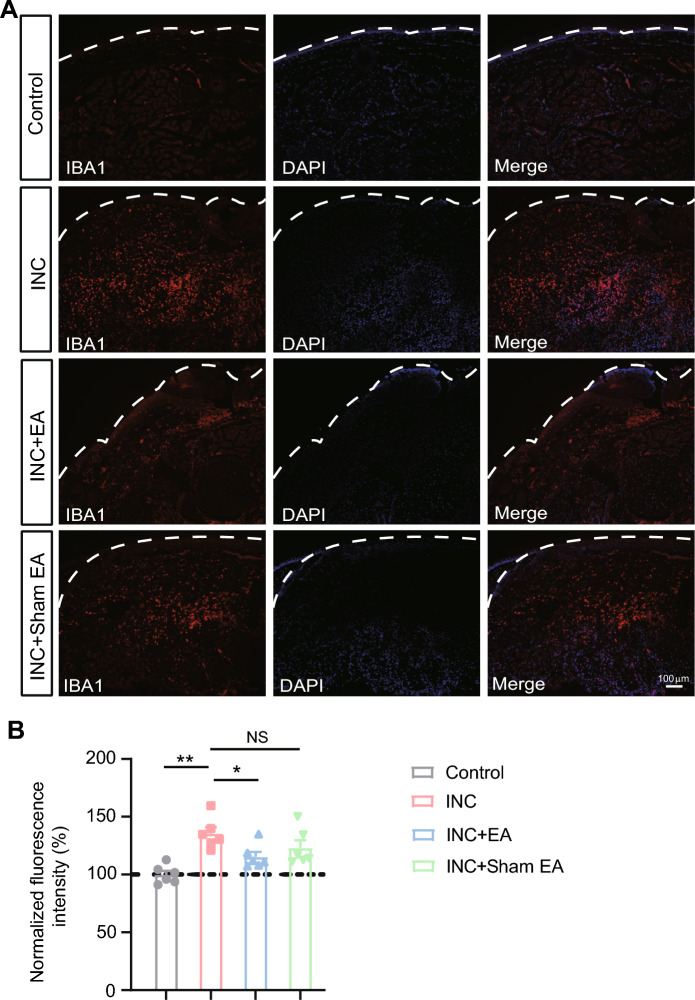
EA intervention decreases macrophage infiltration in the incised skin of incisional pain model mice. **A** Immunofluorescence pictures showing the expression of the macrophage specific marker Iba-1 (red) in skin tissues of control, INC, INC + EA and INC + sham EA group. DAPI was counterstained in purple. **B** Summarized results of the mean fluorescence intensity of Iba-1 immunostaining as in panel A. The fluorescence intensity from the control group was taken as 100% and all other groups were normalized thereafter. n = 6 mice/group. ^*^p < 0.05 and ^**^p < 0.01

Our recent work reveals that IL-33 acts upon ST2 expressed in macrophages to promote macrophage infiltration in incised skin that contributes to incisional pain [12]. Since we found that EA could reduce IL-33 overproduction in the incised skin, we wondered whether EA might affect macrophage infiltration. We then examined the amount of macrophages in the skin via immunostaining using the macrophage marker Iba-1. Immunostaining showed that there was indeed a significant influx of macrophage in the incised skin of incisional pain model mice vs. control mice, a result consistent with our recent study [12] (Fig. 5A, B). EA intervention significantly reduced the infiltration of macrophages in skin tissues of incisional pain model mice. In contrast, sham EA had no obvious effect on macrophage infiltration (Fig. 5A, B).

**Fig. 6:**
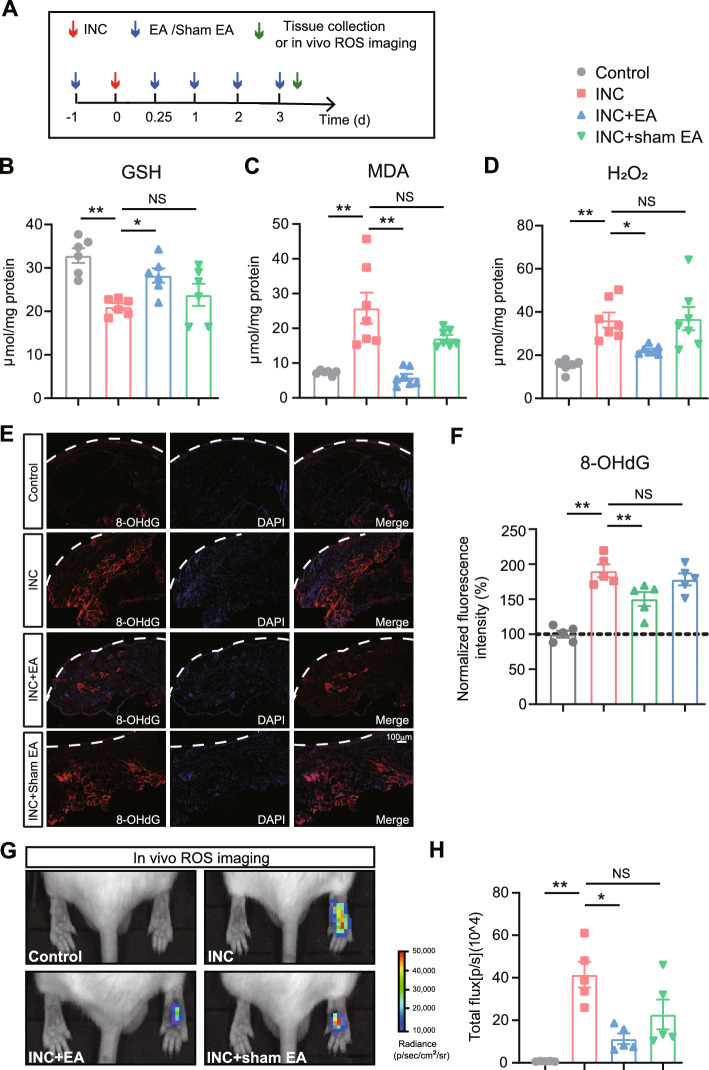
EA intervention reduces the oxidative stress increases in the incised skin tissues of incisional pain model mice. **A** Time schedule for the experiments being done. **B**–**D** Results summary of biochemical assays for GSH-Px, MDA and H_2_O_2_ in skin tissues of control, INC, INC + EA and INC + sham EA groups. n = 6–7 mice/group. **E** Immunostaining of 8-OHdG (in red) in skin tissues of the four groups. **F** Summary of normalized 8-OHdG immunofluorescence intensity in panel **E**. Value from the control group was taken as 100% and all other groups were normalized thereafter. n = 5 mice/group. **G** In vivo ROS imaging of the incised skin area by means of L-012 in four groups of mice. **H** Summary of the in vivo imaging results as in panel G. n = 5 mice/group. ^*^p < 0.05 and ^**^p < 0.01

It is known that infiltrating macrophages can produce ROS through oxidative burst to combat invasive microorganisms in the wounded skin [43]. We recently showed that macrophages constitute a major cellular source for ROS production in the incised skin [12]. Since EA intervention could reduce macrophage infiltration, we proceeded to study whether EA might affect ROS production in the incised skin. After the last EA treatment session on Day 3, the skin tissues of control, INC, INC + EA and INC + sham EA group mice were collected for biochemical assays of oxidative stress (Fig. 6A). We found that the activity of GSH-Px, an important antioxidant enzyme involved in oxidative stress modulation, decreased significantly in skin tissues from INC model mice compared with control mice (Fig. 6B). EA intervention replenished the lost activity of GSH-Px in the incised skin of INC model mice, whereas sham EA had no effect (Fig. 6B). Furthermore, we found that the concentrations of malondialdehyde (MDA), a lipid peroxidation product, as well as hydrogen peroxide (H_2_O_2_), an endogenous ROS product, were both significantly elevated in the incised skin of INC model mice (Fig. 6C, D). After EA intervention, the concertation of MDA and H_2_O_2_ were both significantly reduced. As a comparison, sham EA showed no such effect on MDA and H_2_O_2_ (Fig. 6C, D). In parallel with the above findings, immunostaining revealed that the expression of 8-hydroxydeoxyguanosine (8-OHdG), a biomarker for oxidative damage to DNA, was significantly increased in the incised skin of incisional pain model mice vs. control mice (Fig. 6E, F). EA intervention reduced the overexpression of 8-OHdG in the incised skin. In contrast, sham EA showed no such effect (Fig. 6E, F).

Next, the endogenous ROS production in the incised skin area was further monitored noninvasively using the chemiluminescent probe L-012 via in vivo imaging. In vivo ROS imaging was performed 3 days post-skin incision in live mice. We found that there was a strong increase in L-012 chemiluminescent signal in the incised skin of incisional pain model mice compared with control mice, an indication of in vivo ROS production upon skin incision (Fig. 6E, F). EA intervention significantly reduced the overproduction of ROS in vivo, whereas sham EA did not show obvious ameliorative effect on ROS production (Fig. 6G, H). These findings indicate that EA is capable of ameliorating oxidative stress as well as the related cellular DNA damage in the incised skin tissues of the incisional pain model mice.

### Adoptive transfer of macrophages reversed EA-induced analgesia in incisional pain model mice

**Fig. 7:**
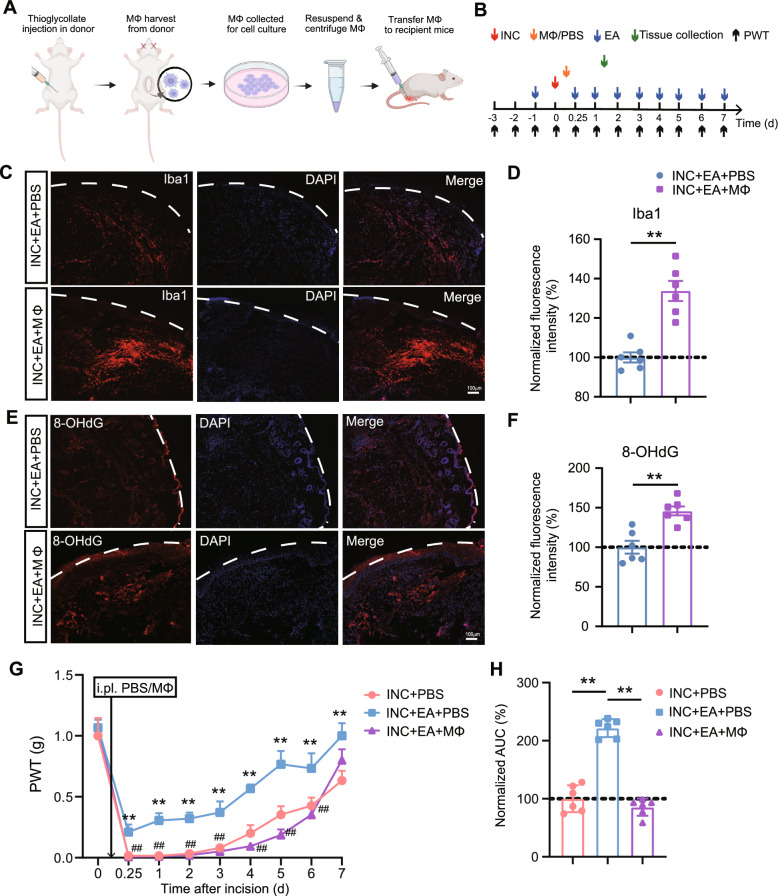
Adoptive transfer of macrophages in the incised skin reverses EA-induced analgesia in incisional pain model mice. **A** Work flow for macrophage collection, culture and the adoptive transfer. **B** Time schedule of all the experiments being included. **C** Iba-1 immunostaining in INC + EA + PBS and INC + EA + Macrophages (Mφs) group of mice. **D** Summary of the normalized fluorescence intensity of Iba-1 as in panel C. ^**^p < 0.01. **E** 8-OHdG immunostaining in INC + EA + PBS and INC + EA + Mφs group of mice. **F** Summary of the normalized fluorescence intensity of Iba-1 as in panel **E**. The fluorescence intensity from the INC + EA + PBS group was taken as 100% and the other group was normalized thereafter. ^**^p < 0.01. Scale bar = 100 μm. Stratum corneum of epidermis is illustrated by the white dashed line. **G** Time courses showing the PWT changes upon Mφs adoptive transfer in mice. ^**^p < 0.01 vs. INC + PBS group. ^##^p < 0.01 vs. INC + EA + PBS group. **H** Summary of normalized AUC analysis of curves in panel **G**. n = 6 mice/group. ^**^p < 0.01

The above results indicate that EA intervention may affect IL-33 signaling-induced macrophage recruitment to the incised skin, resulting in amelioration of ROS production as well as pain responses. We then wondered whether the adoptive transfer of excessive macrophages to the incised skin may reverse these EA-induced ameliorative effects on incisional pain model mice. Mouse peritoneal macrophages (Mφs) were obtained via thioglycollate elicitation (Fig. 7A) [40]. After cell collection and culture, the mouse macrophages were injected into the incised skin tissue 4 h after the incision was made (Fig. 7A, B). EA intervention was applied thereafter as indicated (Fig. 7B). Immunostaining showed that Iba-1 immunofluorescence signal was significantly increased in the incised skin of mice receiving macrophage adoptive transfer (INC + EA + Mφs group) compared with PBS-injected mice (INC + EA + PBS group) (Fig. 7C, D). This result confirmed the successful transfer of macrophages to the incised skin of incisional pain model mice. Next, the oxidative stress in the incised skin was monitored via 8-OHdG immunostaining. As shown in Fig. 7E, F, 8-OHdG immunofluorescence signal was significantly upregulated in the incised skin of INC + EA + Mφs group mice compared with INC + EA + PBS group mice. Furthermore, the adoptive transfer of macrophages significantly reversed the analgesic effect of EA on incisional pain model group mice (INC + EA + Mφs group vs. INC + EA + PBS group) (Fig. 7G, H). Therefore, these results demonstrate that the adoptive transfer of macrophages can reverse EA-induced analgesia in incisional pain model mice.

### EA intervention does not affect skin wound healing process of incisional pain model mice

In the last, we continued to evaluate whether EA intervention might affect the skin wound healing process in the incisional pain model mice. The skin wound healing process was evaluated by measuring the width of the wound with or without EA intervention on a daily basis after the incision was made. As shown in Fig. S1A, B, EA-treated mice did not exhibit any obvious deficit in wound healing capability compared with WT mice after skin incision. This result thus rules out the possibility that EA might affect skin wound healing, furthering suggesting the potential and possibility of EA to be used as an alternative and green therapy for postoperative pain management.

## Discussion

In this study, our work demonstrated that 2/100 Hz EA was effective for ameliorating pain response and improving gait impairments in a mouse model of incisional pain. We then performed RNA-Seq on the incised skin with the aim to explore the potential signaling pathways or genes that EA may affect. We found that EA significantly reduced *Il33* gene overexpression in the incised skin tissues of model mice. Further mechanistic explorations revealed that IL-33 was mainly produced from keratinocytes after skin was incised. EA treatment could lower down IL-33 overproduction from the keratinocytes, thus resulting in less macrophage infiltration and less ROS accumulation in the incised skin area. Adoptive transfer of excessive macrophages into the incised skin abrogated ameliorating effects of EA on macrophage infiltration and ROS accumulation, resulting in the reversion of EA-induced analgesia on incisional pain model mice. At last, we proved that EA intervention did not affect skin wound healing process.

Postoperative pain can significantly affect the living quality and even the rehabilitation of the patients if not managed adequately [1]. Currently, the major pharmacological methods for relieving postoperative pain largely depend on NSAIDs and opioids. However, the frequent usage of these mediations may lead to many side effects, including GI toxicity and addiction, etc. Therefore, the exploration of alternative therapeutic options with less side effects for postoperative pain management is an urgent necessity. Acupuncture has been widely utilized for pain control in clinic. Extensive studies have proved the effectiveness of acupuncture for pain management [44–47]. In regard to postoperative pain management, there are clinical studies proving acupuncture’s effectiveness in relieving postoperative pain and reducing the usage of analgesics in patients [16, 17]. Therefore, acupuncture can be used as an alternative approach for the relief of postoperative pain in clinical practice.

Tissue inflammation is recognized as an important factor mediating postoperative pain. After the surgery, a diverse panel of immune cells are recruited to the injured site. These immune cells secrete a broad spectrum of pro-inflammatory cytokines and chemokines that activate or sensitize nociceptors [48]. Prior studies have shown that EA can modulate local inflammation, resulting in pain amelioration of certain inflammatory pain conditions [23, 26, 49]. In addition, recent work from us also demonstrated that EA can intervene with neuro-immune interaction in periphery or spinal dorsal horn to reduce neuroinflammation and relieve pain [24, 50, 51]. To gain a more comprehensive overview about how EA might affect local inflammation in the incised skin, we thus performed RNA-Seq with the aim to explore potential signaling pathways or genes that EA may affect. Bioinformatics analysis suggests that EA treatment can significantly downregulate cytokine-cytokine receptor pathway in the incised skin. Within this pathway, we found *Il33* gene appeared as one of the most significantly upregulated cytokine genes in incisional pain group vs. control group, an elevation that was also suppressed following EA intervention.

IL-33, a cytokine from the IL-1 family, signals through the receptor complex comprising ST2 and IL-1 receptor accessory protein to modulate inflammation, autoimmune responses, and homeostasis [52]. Beyond its classical role in immune modulation, IL-33 gradually emerges as a crucial mediator in pain and itch pathogenesis [53–55]. Our recent work identified that IL-33 is significantly increased in keratinocytes of the incised skin of incisional pain model mice. It can be released into extracellular space to attract macrophage infiltration via ST2-dependent mechanism into the incised skin. Infiltrated macrophages then produce large amount of ROS to activate nociceptive TRPA1 channel innervating the incisional skin, which results in pain generation [12]. Additionally, the modulatory effect of EA on IL-33 expression in pain model animals has been documented by some prior studies. It is reported that EA can reduce IL-33 overexpression in spinal cord tissues of animal models of formalin-induced acute pain and acute to chronic pain transition model [56, 57]. Based upon these previous findings, we decided to explore whether EA may affect IL-33 signaling aforementioned to produce analgesic effect on incisional pain model mice. We found that IL-33 was predominantly produced from keratinocytes in the incised skin from both incisional to proximal and proximal to distal area. This observation is consistent with our recent study [12]. Based upon this observation, we further found that EA intervention could significantly reduce the overexpression of IL-33 in keratinocytes of the incised skin. Quantification by immunoblotting confirmed the immunostaining result of EA-induced IL-33 expression modulation in incised skin tissues. Therefore, this result indicates that EA is capable of modulating IL-33 expression in the incisional skin of the incisional pain model mice.

It is well known that macrophage infiltration make an important contributions to pain pathophysiology [58–61]. IL-33 can directly or indirectly attract macrophages through activating ST2 receptor-mediated cellular signaling [12, 62, 63]. Since we found EA could attenuate IL-33 overproduction, we then wanted to know whether this would result in less macrophage infiltration in the incised skin. Our results indicate that EA treatment attenuated macrophage infiltration in the incised skin of incisional pain model mice. Locally infiltrated macrophages can produce significant amount of reactive oxygen species (ROS). These molecules are crucial for promoting wound healing process as well as for pathogen control in the incised site [64]. However, excessive accumulation of ROS may also result in detrimental effects, including tissue inflammation and incisional pain [12, 64–66]. ROS can activate TRPA1 channel expressed in nerve endings to trigger pain or cause peripheral sensitization that exacerbates pain [23, 59]. Our recent work demonstrated that infiltrated macrophages in the incised skin release large amount of ROS that contribute to the incisional pain [12]. Therefore, we continued to examine whether EA could attenuate ROS accumulation in the incised skin. Our result indicated that EA treatment could significantly reduce excessive ROS production in the incised skin. In vivo ROS imaging further confirmed the effects of EA on ROS production in live animals with skin incision.

Since EA treatment can reduce the infiltration of macrophages in the incised skin, we then wondered whether the transfer of extra amount of macrophages may reverse EA-induced ameliorative effects on incisional pain model mice. In our previous study, the transfer of macrophages from wild type mice could restore the oxidative injury in the incised tissue and reproduce pain responses in *St2*^−/−^ mice after skin was incised [12]. Our results first confirmed the successful transfer of mouse naïve macrophages and their retaining in the incised skin tissues after EA treatment. The transfer of macrophages significantly boosted oxidative stress-related cellular DNA damage in the incised skin of EA-treated mice and further reversed EA-induced analgesia in incisional pain model mice. Therefore, these results in all indicate that EA can reduce IL-33 overexpression in local incised skin tissues, resulting in less macrophage infiltration and ROS production, all of which contribute to EA-induced analgesic effect on incisional pain model mice.

In order to rule out the possibility that EA may affect the skin wound healing process, we decided to examine the effects of EA treatment on skin wound healing. Our results indicate that EA-treated mice did not exhibit any significant deficiency in wound healing process vs. non-treated mice. Actually, it has been found that acupuncture can accelerate wound healing process via mechanisms involving the promotion of re-epithelialization and angiogenesis as well as amelioration of inflammation [67–69]. Therefore, the effect of blocking ROS on wound healing may be balanced by aforementioned beneficial effects of EA on wound healing. In addition, we found EA treatment did not totally abolish ROS generation in the incised skin. The residual ROS in the incised tissue after EA intervention may be sufficient enough for serving its role in promoting wound healing.

It should come to attention that certain additional chemokines or signaling molecules may also contribute to macrophage infiltration mechanism besides the IL-33 signaling that we especially focused in this study. Actually, our RNA-Seq identified a panel of cytokines that are upregulated in the incision, whereas downregulated by EA treatment. Among these cytokines that are responsive to EA treatment, CXCL10 has been found to be involved in macrophages infiltration and can cause pain via producing neuroinflammation in the spinal cord of a mouse model of experimental autoimmune prostatitis [70]. Another important cytokine that is responsive to EA treatment we identified through RNA-Seq is TNF-α. TNF-α has been found to be highly upregulated in the mouse incisional pain model in prior study [71]. Furthermore, blocking TNF-α signaling can reduce pain response and further downregulate *Nav1.8* and *Nav1.9* gene expression in DRG neurons of incisional pain model mice, indicating an important participation of TNF-α in the pathogenesis of incisional pain via modulating nociceptive ion channels [71]. In our hands, the RNA-Seq experiments identified that *Tnfa* gene were indeed upregulated in the incised skin, a result consistent with that prior study. We further found EA treatment could significantly downregulate *Tnfa* gene upregulation. These findings suggest that in addition to IL-33 signaling, EA may also affect other pain-related signaling in the incised skin, reflecting acupuncture as a multi-targeted physiotherapy for pain management. Further studies will be needed to confirm the above findings from the RNA-Seq exploration.

## Conclusions

Our present work demonstrates that EA can ameliorate incisional pain via mechanisms involving suppression of IL-33 overproduction in the incised skin tissues. EA-induced suppression of IL-33 overproduction results in less macrophage infiltration and ROS production in the incised skin tissues, which contributes to the analgesic effect. Our work provides novel mechanistic insights into the understanding of EA’s analgesia on postoperative pain and further supports EA to be used as an alternative and green therapy for postoperative pain management.

## Supplementary Information

Supplementary material 1: Figure S1. EA intervention does not affect skin wound healing process.

## Data Availability

The key data are contained in the manuscript. Further request can be obtained from the corresponding author Dr. Boyi Liu.

## Abbreviations

8-OHdG: 8-hydroxydeoxyguanosine
AUC: Area under the curve
CB1R: Cannabinoid receptor type 1
CPS: Allopurinol
CT: Cycle threshold
DEGs: Differentially expressed genes
DRG: Dorsal root ganglion
EA: Electroacupuncture
GI: Gastrointestinal
GLT-1: Glutamate transporter-1
GSH-Px: Glutathione peroxidase
INC: Incisional
Krt14: Keratin 14
Mφs: Macrophages
MDA: Malondialdehyde
NSAIDs: Nonsteroidal anti-inflammatory drugs
PWT: Paw withdrawal threshold
RNA-Seq: RNA-Sequencing
ROS: Reactive oxygen species
SOD: Superoxide dismutase
TPMs: Thioglycollate-elicited macrophages

## References

[1] Liu Y, Xiao S, Yang H, Lv X, Hou A, Ma Y, et al. Postoperative pain-related outcomes and perioperative pain management in China: a population-based study. Lancet Reg Health. 2023;39:100822. 10.1016/j.lanwpc.2023.100822. 10.1016/j.lanwpc.2023.100822 PMC10625022 37927993

[2] Small C, Laycock H. Acute postoperative pain management. Br J Surg. 2020;107(2):e70–80. 10.1002/bjs.11477. 31903595 10.1002/bjs.11477

[3] Apfelbaum JL, Chen C, Mehta SS, Gan TJ. Postoperative pain experience: results from a national survey suggest postoperative pain continues to be undermanaged. Anesth Analg. 2003;97(2):534–40. 10.1213/01.ANE.0000068822.10113.9E. 12873949 10.1213/01.ANE.0000068822.10113.9E

[4] Colvin LA, Bull F, Hales TG. Perioperative opioid analgesia-when is enough too much? A review of opioid-induced tolerance and hyperalgesia. Lancet. 2019;393(10180):1558–68. 10.1016/S0140-6736(19)30430-1. 30983591 10.1016/S0140-6736(19)30430-1

[5] Macintyre PE, Quinlan J, Levy N, Lobo DN. Current issues in the use of opioids for the management of postoperative pain: a review. JAMA Surg. 2022;157:158–66. 34878527 10.1001/jamasurg.2021.6210

[6] Wick EC, Grant MC, Wu CL. Postoperative multimodal analgesia pain management with nonopioid analgesics and techniques: a review. JAMA Surg. 2017;152:691–7. 28564673 10.1001/jamasurg.2017.0898

[7] Pogatzki-Zahn E, Segelcke D, Zahn P. Mechanisms of acute and chronic pain after surgery: update from findings in experimental animal models. Curr Opin Anaesthesiol. 2018;31(5):575–85. 10.1097/ACO.0000000000000646. 30028733 10.1097/ACO.0000000000000646

[8] Xu R, Wang J, Nie H, Zeng D, Yin C, Li Y, et al. Genome-wide expression profiling by RNA-sequencing in spinal cord dorsal horn of a rat chronic postsurgical pain model to explore potential mechanisms involved in chronic pain. J Pain Res. 2022;15:985–1001. 35411184 10.2147/JPR.S358942 PMC8994637

[9] Shankar Hari M, Summers C. Major surgery and the immune system: from pathophysiology to treatment. Curr Opin Crit Care. 2018;24(6):588–93. 10.1097/MCC.0000000000000561. 30299310 10.1097/MCC.0000000000000561

[10] Larouche J, Sheoran S, Maruyama K, Martino MM. Immune regulation of skin wound healing: mechanisms and novel therapeutic targets. Adv Wound Care. 2018;7(7):209–31. 10.1089/wound.2017.0761. 10.1089/wound.2017.0761 PMC6032665 29984112

[11] Wu C, Erickson MA, Xu J, Wild KD, Brennan TJ. Expression profile of nerve growth factor after muscle incision in the rat. Anesthesiology. 2009;110:140–9. 19104181 10.1097/ALN.0b013e318190bc84 PMC2727137

[12] Xu R, Pan Y, Zheng K, Chen M, Yin C, Hu Q, et al. IL-33/ST2 induces macrophage-dependent ROS production and TRPA1 activation that mediate pain-like responses by skin incision in mice. Theranostics. 2024;14:5281–302. 39267790 10.7150/thno.97856 PMC11388077

[13] McEwan TB, Sophocleous RA, Cuthbertson P, Mansfield KJ, Sanderson-Smith ML, Sluyter R. Autocrine regulation of wound healing by ATP release and P2Y(2) receptor activation. Life Sci. 2021;283:119850. 10.1016/j.lfs.2021.119850. 34314735 10.1016/j.lfs.2021.119850

[14] Liu B, Chen B, Guo Y, Tian L. Acupuncture – a national heritage of China to the world: international clinical research advances from the past decade. Acupunct Herb Med. 2021;1(2):65–73. 10.1097/HM9.0000000000000017.

[15] Balgis HS, Wiyono N. Electroacupuncture for pain therapy: a bibliometric analysis and content review update for 1 decade (2013-2022). Med Acupunct. 2024;36:189–202. 39668854 10.1089/acu.2023.0083 PMC11632150

[16] Liu J, Li Y, Liu J, Zhang X. Efficacy of acupuncture in postoperative pain-relieving: a systematic review and meta-analysis. Pain Manag Nurs. 2025;26(3):319–29. 10.1016/j.pmn.2024.12.014. 39814622 10.1016/j.pmn.2024.12.014

[17] Wu MS, Chen KH, Chen IF, Huang SK, Tzeng PC, Yeh ML, et al. The efficacy of acupuncture in post-operative pain management: a systematic review and meta-analysis. PLoS ONE. 2016;11(3):e0150367. 10.1371/journal.pone.0150367. 26959661 10.1371/journal.pone.0150367 PMC4784927

[18] Wang JY, Zhang JL, Chen SP, Gao YH, Zhang JL, Chen Y, et al. Electroacupuncture relieves hyperalgesia by regulating neuronal-glial interaction and glutamate transporters of spinal dorsal horns in rats with acute incisional neck pain. Front Neurosci. 2022;16:885107. 10.3389/fnins.2022.885107. 36389227 10.3389/fnins.2022.885107 PMC9643735

[19] Wang J, Zhang J, Gao Y, Chen Y, Duanmu C, Liu J. Electroacupuncture alleviates hyperalgesia by regulating CB1 receptor of spinal cord in incisional neck pain rats. Evid Based Complement Alternat Med. 2021;2021:5880690. 10.1155/2021/5880690. 34961820 10.1155/2021/5880690 PMC8710158

[20] Wang JY, Bai WZ, Gao YH, Zhang JL, Duanmu CL, Liu JL. GABAergic inhibition of spinal cord dorsal horns contributes to analgesic effect of electroacupuncture in incisional neck pain rats. J Pain Res. 2020;13:1629–45. 10.2147/JPR.S242330. 32694919 10.2147/JPR.S242330 PMC7340366

[21] Wang JY, Gao YH, Qiao LN, Zhang JL, Rong PJ, Liu JL. Electroacupuncture alleviated incisional neck pain possibly by suppressing TNF-α expression and increasing IL-4/IL-4 receptor signaling in cervical dorsal part of spinal cord in incisio-nal neck pain rats. Zhen Ci Yan Jiu. 2019;44:703–8. 31657158 10.13702/j.1000-0607.190355

[22] Chu WG, Zhang R, Li HT, Li YC, Ding H, Li ZZ, et al. Locus coeruleus noradrenergic-spinal projections contribute to electroacupuncture-mediated antinociception in postoperative pain in mice. Adv Sci. 2025. 10.1002/advs.202501182. 10.1002/advs.202501182 PMC12224967 40387368

[23] Li X, Yin C, Hu Q, Wang J, Nie H, Liu B, et al. Nrf2 activation mediates antiallodynic effect of electroacupuncture on a rat model of complex regional pain syndrome type-I through reducing local oxidative stress and inflammation. Oxid Med Cell Longev. 2022;2022(1):8035109. 10.1155/2022/8035109. 35498128 10.1155/2022/8035109 PMC9054487

[24] Wei H, Liu B, Yin C, Zeng D, Nie H, Li Y, et al. Electroacupuncture improves gout arthritis pain via attenuating ROS-mediated NLRP3 inflammasome overactivation. Chin Med. 2023;18:86. 37464384 10.1186/s13020-023-00800-1 PMC10355064

[25] Yu ML, Wei RD, Zhang T, Wang JM, Cheng Y, Qin FF, et al. Electroacupuncture relieves pain and attenuates inflammation progression through inducing IL-10 production in CFA-induced mice. Inflammation. 2020;43:1233–45. 32198725 10.1007/s10753-020-01203-2

[26] Zhang Y, Wang H, Gong YN, Yang FM, Wang SJ, Liu YY, et al. Pathological pathway analysis in an experimental rheumatoid arthritis model and the tissue repair effect of acupuncture at ST36. Front Immunol. 2023;14:1164157. 37256145 10.3389/fimmu.2023.1164157 PMC10225595

[27] Barabas ME, Stucky CL. TRPV1, but not TRPA1, in primary sensory neurons contributes to cutaneous incision-mediated hypersensitivity. Mol Pain. 2013;9:9. 10.1186/1744-8069-9-9. 23497345 10.1186/1744-8069-9-9 PMC3602024

[28] Liu B, Fan L, Balakrishna S, Sui A, Morris JB, Jordt SE. TRPM8 is the principal mediator of menthol-induced analgesia of acute and inflammatory pain. Pain. 2013;154:2169–77. 23820004 10.1016/j.pain.2013.06.043 PMC3778045

[29] Zheng X, Tai Y, He D, Liu B, Wang C, Shao X, et al. ETAR and protein kinase A pathway mediate ET-1 sensitization of TRPA1 channel: a molecular mechanism of ET-1-induced mechanical hyperalgesia. Mol Pain. 2019;15:1744806919842473. 30990108 10.1177/1744806919842473 PMC6537062

[30] Brennan TJ, Vandermeulen EP, Gebhart GF. Characterization of a rat model of incisional pain. Pain. 1996;64:493–502. 8783314 10.1016/0304-3959(95)01441-1

[31] Chai W, Tai Y, Shao X, Liang Y, Zheng GQ, Wang P, et al. Electroacupuncture alleviates pain responses and inflammation in a rat model of acute gout arthritis. Evid Based Complement Alternat Med. 2018;2018(1):2598975. 10.1155/2018/2598975. 29743920 10.1155/2018/2598975 PMC5884439

[32] Ren J, Li N, Xi D, Wei N, Shao X, Liu B, et al. Role of mast cell in hyperalgesic priming and the preventive effect of electroacupuncture on the transition from acute to chronic pain. Acupunct Herb Med. 2024;4(4):525–37. 10.1097/HM9.0000000000000140.

[33] Zeng D, Yin C, Wei H, Li Y, Yang Y, Nie H, et al. Activation of Nrf2 antioxidant signaling alleviates gout arthritis pain and inflammation. Biomed Pharmacother. 2024;170:115957. 10.1016/j.biopha.2023.115957. 38042115 10.1016/j.biopha.2023.115957

[34] Wang J, Yin C, Pan Y, Yang Y, Li W, Ni H, et al. CXCL13 contributes to chronic pain of a mouse model of CRPS-I via CXCR5-mediated NF-κB activation and pro-inflammatory cytokine production in spinal cord dorsal horn. J Neuroinflammation. 2023;20:109. 37158939 10.1186/s12974-023-02778-x PMC10165831

[35] Yin C, Liu B, Dong Z, Shi S, Peng C, Pan Y, et al. CXCL5 activates CXCR2 in nociceptive sensory neurons to drive joint pain and inflammation in experimental gouty arthritis. Nat Commun. 2024;15:3263. 38627393 10.1038/s41467-024-47640-7 PMC11021482

[36] Yin C, Hu Q, Liu B, Tai Y, Zheng X, Li Y, et al. Transcriptome profiling of dorsal root ganglia in a rat model of complex regional pain syndrome type-I reveals potential mechanisms involved in pain. J Pain Res. 2019;12:1201–16. 31114302 10.2147/JPR.S188758 PMC6489655

[37] Livak KJ, Schmittgen TD. Analysis of relative gene expression data using real-time quantitative PCR and the 2(-Delta Delta C(T)) method. Methods. 2001;25(4):402–8. 10.1006/meth.2001.1262. 11846609 10.1006/meth.2001.1262

[38] Liu B, Tai Y, Liu B, Caceres AI, Yin C, Jordt SE. Transcriptome profiling reveals Th2 bias and identifies endogenous itch mediators in poison ivy contact dermatitis. JCI Insight. 2019;5:e124497. 31184997 10.1172/jci.insight.124497 PMC6675552

[39] Yin C, Liu B, Wang P, Li X, Li Y, Zheng X, et al. Eucalyptol alleviates inflammation and pain responses in a mouse model of gout arthritis. Br J Pharmacol. 2020;177(9):2042–57. 10.1111/bph.14967. 31883118 10.1111/bph.14967 PMC7161556

[40] Yin C, Liu B, Li Y, Li X, Wang J, Chen R, et al. IL-33/ST2 induces neutrophil-dependent reactive oxygen species production and mediates gout pain. Theranostics. 2020;10:12189–203. 33204337 10.7150/thno.48028 PMC7667675

[41] Li Y, Yin C, Li X, Liu B, Wang J, Zheng X, et al. Electroacupuncture alleviates paclitaxel-induced peripheral neuropathic pain in rats via suppressing TLR4 signaling and TRPV1 upregulation in sensory neurons. Int J Mol Sci. 2019. 10.3390/ijms20235917. 31775332 10.3390/ijms20235917 PMC6929119

[42] Arnstein P, van Boekel R, Booker SQ. CE: overcoming movement-evoked pain to facilitate postoperative recovery. Am J Nurs. 2023;123(7):28–37. 10.1097/01.NAJ.0000944916.30662.5c. 37345778 10.1097/01.NAJ.0000944916.30662.5c PMC10830148

[43] Krzyszczyk P, Schloss R, Palmer A, Berthiaume F. The role of macrophages in acute and chronic wound healing and interventions to promote pro-wound healing phenotypes. Front Physiol. 2018;9:419. 29765329 10.3389/fphys.2018.00419 PMC5938667

[44] Vickers AJ, Vertosick EA, Lewith G, MacPherson H, Foster NE, Sherman KJ, et al. Acupuncture for chronic pain: update of an individual patient data meta-analysis. J Pain. 2018;19:455–74. 29198932 10.1016/j.jpain.2017.11.005 PMC5927830

[45] Wan K, Xu Q, Shi Y, Cui C, Lei J, Zhang K, et al. Electroacupuncture produces analgesic effects via cannabinoid CB1 receptor-mediated GABAergic neuronal inhibition in the rostral ventromedial medulla. Chin Med. 2025;20:30. 40038719 10.1186/s13020-025-01083-4 PMC11881457

[46] Chen Z, Yao K, Wang X, Liu Y, Du S, Wang S, et al. Acupuncture promotes muscle cells ATP metabolism in ST36 acupoint local exerting effect by activating TRPV1/CaMKII/AMPK/PGC1α signaling pathway. Chin Med. 2025;20:112. 40660305 10.1186/s13020-025-01169-z PMC12257683

[47] Ma X, Chen W, Fu Y, Li H, Liu C. Acupuncture for neuropathic pain: focusing on the sympathetic nerve system. Acupunct Herb Med. 2023;3(3):139–48. 10.1097/HM9.0000000000000069.

[48] Zheng K, Chen M, Xu X, Li P, Yin C, Wang J, et al. Chemokine CXCL13-CXCR5 signaling in neuroinflammation and pathogenesis of chronic pain and neurological diseases. Cell Mol Biol Lett. 2024;29:134. 39472796 10.1186/s11658-024-00653-y PMC11523778

[49] Chen Z, Wang X, Du S, Yao K, Guo Y, Lin X. Acupuncture at the Zusanli acupoint can reduce the inflammatory response in AIA mice by regulating the arachidonic acid and pentose phosphate pathways. J Chromatogr B Analyt Technol Biomed Life Sci. 2024;1247:124307. 10.1016/j.jchromb.2024.124307. 39306868 10.1016/j.jchromb.2024.124307

[50] Zhang Y, Chen R, Hu Q, Wang J, Nie H, Yin C, et al. Electroacupuncture ameliorates mechanical allodynia of a rat model of CRPS-I via suppressing NLRP3 inflammasome activation in spinal cord dorsal horn neurons. Front Cell Neurosci. 2022;16:826777. 10.3389/fncel.2022.826777. 35693886 10.3389/fncel.2022.826777 PMC9174662

[51] Li Y, Xu R, Chen M, Zheng K, Nie H, Yin C, et al. Electroacupuncture alleviates paclitaxel-induced peripheral neuropathy by reducing CCL2-mediated macrophage infiltration in sensory ganglia and sciatic nerve. Chin Med. 2025;20:9. 39806462 10.1186/s13020-024-01023-8 PMC11727193

[52] Liew FY, Girard JP, Turnquist HR. Interleukin-33 in health and disease. Nat Rev Immunol. 2016;16:676–89. 27640624 10.1038/nri.2016.95

[53] Li P, Yu Q, Nie H, Yin C, Liu B. IL-33/ST2 signaling in pain and itch: cellular and molecular mechanisms and therapeutic potentials. Biomed Pharmacother. 2023;165:115143. 10.1016/j.biopha.2023.115143. 37450998 10.1016/j.biopha.2023.115143

[54] Fattori V, Hohmann MSN, Rossaneis AC, Manchope MF, Alves-Filho JC, Cunha TM, et al. Targeting IL-33/ST2 signaling: regulation of immune function and analgesia. Expert Opin Ther Targets. 2017;21(12):1141–52. 10.1080/14728222.2017.1398734. 29076792 10.1080/14728222.2017.1398734

[55] Liu B, Tai Y, Achanta S, Kaelberer MM, Caceres AI, Shao X, et al. IL-33/ST2 signaling excites sensory neurons and mediates itch response in a mouse model of poison ivy contact allergy. Proc Natl Acad Sci U S A. 2016;113(47):E7572–9. 10.1073/pnas.1606608113. 27821781 10.1073/pnas.1606608113 PMC5127381

[56] Jin Y, Zhou J, Fang Y, Song H, Lin S, Pan B, et al. Electroacupuncture prevents the development or establishment of chronic pain via IL-33/ST2 signaling in hyperalgesic priming model rats. Neurosci Lett. 2024;820:137611. 10.1016/j.neulet.2023.137611. 38142925 10.1016/j.neulet.2023.137611

[57] Han P, Liu S, Zhang M, Zhao J, Wang Y, Wu G, et al. Inhibition of spinal interlukin-33/ST2 signaling and downstream ERK and JNK pathways in electroacupuncture analgesia in formalin mice. PLoS ONE. 2015;10(6):e0129576. 10.1371/journal.pone.0129576. 26067287 10.1371/journal.pone.0129576 PMC4466274

[58] Chen L, Zou X, Liu CC, Yan P, Deng J, Wang C, et al. Earlier onset of chemotherapy-induced neuropathic pain in females by ICAM-1-mediated accumulation of perivascular macrophages. Sci Adv. 2025;11:eadu2159. 40238872 10.1126/sciadv.adu2159 PMC12002127

[59] Titiz M, Landini L, de Souza Monteiro Araujo D, Marini M, Seravalli V, Chieca M, et al. Schwann cell C5aR1 co-opts inflammasome NLRP1 to sustain pain in a mouse model of endometriosis. Nat Commun. 2024;15:10142. 39587068 10.1038/s41467-024-54486-6 PMC11589863

[60] Chen O, Donnelly CR, Ji RR. Regulation of pain by neuro-immune interactions between macrophages and nociceptor sensory neurons. Curr Opin Neurobiol. 2020;62:17–25. 31809997 10.1016/j.conb.2019.11.006 PMC7266706

[61] Nie H, Liu B, Yin C, Dong Z, Pan Y, Li P, et al. Neuronal Reg3β/macrophage TNF-α-mediated positive feedback signaling contributes to pain chronicity in a rat model of CRPS-I. Sci Adv. 2025;11:eadu4270. 40749060 10.1126/sciadv.adu4270 PMC12315989

[62] Verri WA Jr., Souto FO, Vieira SM, Almeida SC, Fukada SY, Xu D, et al. IL-33 induces neutrophil migration in rheumatoid arthritis and is a target of anti-TNF therapy. Ann Rheum Dis. 2010;69(9):1697–703. 10.1136/ard.2009.122655. 20472598 10.1136/ard.2009.122655

[63] Yang Y, Andersson P, Hosaka K, Zhang Y, Cao R, Iwamoto H, et al. The PDGF-BB-SOX7 axis-modulated IL-33 in pericytes and stromal cells promotes metastasis through tumour-associated macrophages. Nat Commun. 2016;7:11385. 27150562 10.1038/ncomms11385 PMC4859070

[64] Dunnill C, Patton T, Brennan J, Barrett J, Dryden M, Cooke J, et al. Reactive oxygen species (ROS) and wound healing: the functional role of ROS and emerging ROS-modulating technologies for augmentation of the healing process. Int Wound J. 2017;14(1):89–96. 10.1111/iwj.12557. 26688157 10.1111/iwj.12557 PMC7950185

[65] Sugiyama D, Kang S, Brennan TJ. Muscle reactive oxygen species (ROS) contribute to post-incisional guarding via the TRPA1 receptor. PLoS ONE. 2017;12(1):e0170410. 10.1371/journal.pone.0170410. 28103292 10.1371/journal.pone.0170410 PMC5245866

[66] Yin C, Lyu Q, Dong Z, Liu B, Zhang K, Liu Z, et al. Well-defined alginate oligosaccharides ameliorate joint pain and inflammation in a mouse model of gouty arthritis. Theranostics. 2024;14:3082–103. 38855180 10.7150/thno.95611 PMC11155397

[67] Abali AE, Cabioglu T, Bayraktar N, Ozdemir BH, Moray G, Haberal M. Efficacy of acupuncture on pain mechanisms, inflammatory responses, and wound healing in the acute phase of major burns: an experimental study on rats. J Burn Care Res. 2022;43(2):389–98. 10.1093/jbcr/irab142. 34309681 10.1093/jbcr/irab142

[68] Park SI, Sunwoo YY, Jung YJ, Chang WC, Park MS, Chung YA, et al. Therapeutic effects of acupuncture through enhancement of functional angiogenesis and granulogenesis in rat wound healing. Evid Based Complement Alternat Med. 2012;2012:464586. 10.1155/2012/464586. 23304201 10.1155/2012/464586 PMC3529882

[69] Ishak A, Jusuf AA, Simadibrata CL, Barasila AC, Novita R. Effect of manual acupuncture and laser acupuncture on wound closure in rat with deep partial thickness burn injury. Med Acupunct. 2022;34:240–50. 36051408 10.1089/acu.2021.0083 PMC9419944

[70] Chen L, Chen Z, Chen J, Du H, Chen X, Chen J, et al. CXCL10 promotes spinal macrophage recruitment via the JAK/STAT3 pathway to induce pain in experimental autoimmune prostatitis. Cell Prolif. 2025;58(4):e13784. 10.1111/cpr.13784. 39718951 10.1111/cpr.13784 PMC11969258

[71] de Lima FO, Lauria PSS, do Espírito-Santo RF, Evangelista AF, Nogueira TMO, Araldi D, et al. Unveiling targets for treating postoperative pain: the role of the TNF-α/p38 MAPK/NF-κB/Nav1.8 and Nav1.9 pathways in the mouse model of incisional pain. Int J Mol Sci. 2022. 10.3390/ijms231911630. 36232927 10.3390/ijms231911630 PMC9570460

