# Supplementary material for: Electroacupuncture ameliorates incisional pain via suppressing IL-33 signaling-related macrophage infiltration and ROS overproduction in incised skin
Source: Chin Med. 2026 Jan 9;21:26. doi: 10.1186/s13020-025-01273-0 (PMC12784544; doi:10.1186/s13020-025-01273-0)
Supplement: Supplementary file 1 — Supplementary material 1: Figure S1. EA intervention does not affect skin wound healing process. [file 13020_2025_1273_MOESM1_ESM.docx]

**Supplementary figure**

**
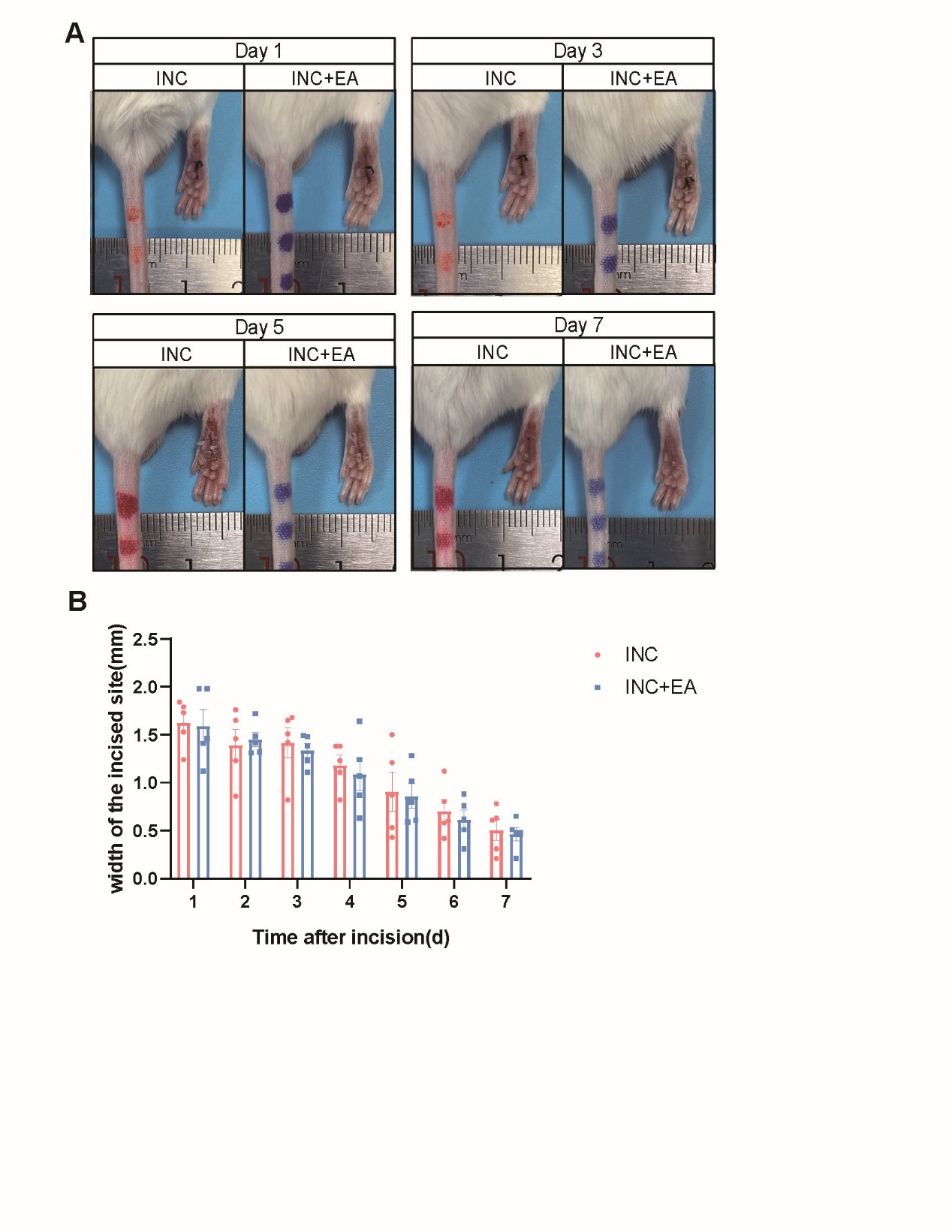
**

**Fig. S1. EA intervention does not affect skin wound healing process.** (A) Representative photos showing the skin wound healing process of the incisional pain model mice with or without EA intervention. Pictures were taken on Day 1, 3, 5 and 7 after the skin was incised. (B) Summary of the width of skin incision (in mm) of INC and INC+EA group of mice, as depicted in panel A. n=5 mice/group.
